# Offspring of Mice Exposed to a Low-Protein Diet in Utero Demonstrate Changes in mTOR Signaling in Pancreatic Islets of Langerhans, Associated with Altered Glucagon and Insulin Expression and a Lower β-Cell Mass

**DOI:** 10.3390/nu11030605

**Published:** 2019-03-12

**Authors:** Renee King, Jessica L. Hill, Bibek Saha, Yuzhen Tong, Brenda J. Strutt, Mark A. Russell, Noel G. Morgan, Sarah J. Richardson, David J. Hill

**Affiliations:** 1 Lawson Health Research Institute, London, ON N6A 4V2, Canada, reneeking@rogers.com (R.K.); jh980@exeter.ac.uk (J.L.H.); bsaha2@uwo.ca (B.S.); ytong33@uwo.ca (Y.T.); brenda.strutt@lawsonresearch.com (B.J.S.); 2 Life Sciences Program, School of Interdisciplinary Science, McMaster University, Hamilton, ON L8S 4LD, Canada; 3 Institute of Biomedical & Clinical Science, University of Exeter Medical School, Exeter EX2 5DW, UK; M.Russell@exeter.ac.uk (M.A.R.); N.G.Morgan@exeter.ac.uk (N.G.M.) S.Richardson@exeter.ac.uk (S.J.R); 4 Department of Physiology and Pharmacology, Western University, London, ON N6A 3K7, Canada; 5 Department of Medicine, Western University, London, ON N6A 3K7, Canada

**Keywords:** β-cell, mTOR, insulin, pancreas, mouse, low-protein, glucagon, STING

## Abstract

Low birth weight is a risk factor for gestational and type 2 diabetes (T2D). Since mammalian target of rapamycin (mTOR) controls pancreatic β-cell mass and hormone release, we hypothesized that nutritional insult in utero might permanently alter mTOR signaling. Mice were fed a low-protein (LP, 8%) or control (C, 20%) diet throughout pregnancy, and offspring examined until 130 days age. Mice receiving LP were born 12% smaller and β-cell mass was significantly reduced throughout life. Islet mTOR levels were lower in LP-exposed mice and localized predominantly to α-rather than β-cells. Incubation of isolated mouse islets with rapamycin significantly reduced cell proliferation while increasing apoptosis. mRNA levels for mTORC complex genes mTOR, Rictor and Raptor were elevated at 7 days in LP mice, as were the mTOR and Raptor proteins. Proglucagon gene expression was similarly increased, but not insulin or the immune/metabolic defense protein STING. In human and mouse pancreas STING was strongly associated with islet β-cells. Results support long-term changes in islet mTOR signaling in response to nutritional insult in utero, with altered expression of glucagon and insulin and a reduced β-cell mass. This may contribute to an increased risk of gestational or type 2 diabetes.

## 1. Introduction

Dietary restriction during human pregnancy results in a reduction in birth weight [1], leading to permanent changes in organ development including the endocrine pancreas [2], and contributing to an adult predisposition to type 2 diabetes and cardio-vascular disease [3]. We and others have previously shown using an isocaloric model of dietary protein restriction (low-protein (LP)) during pregnancy in rodents that dietary insufficiency in early life alters normal pancreatic development, and ultimately contributes to impaired glucose homeostasis in adulthood [4,5,6,7,8,9]. The dietary insult resulted in reduced β-cell mass in the offspring due to a lower β-cell proliferation rate and a greater incidence of apoptosis, the latter seen under basal conditions as well as following exposure to cytotoxic cytokines [4,9]. Islets from LP-fed dams also exhibit long-term impairment of glucose-stimulated insulin release [8,9]. Animals became glucose intolerant by 130 days of age due also to the contribution of peripheral insulin resistance [8].

We utilized the LP diet model to study the development of mouse offspring and found that the β-cell regeneration that normally occurred in juveniles following streptozotocin treatment was prevented if offspring were previously exposed to LP diet [10]. Recently we and others also observed in mice that offspring of LP-fed dams were unable to adequately undergo the adaptive change in β-cell mass that normally occurs during pregnancy, resulting in impaired glucose tolerance during the final week of gestation [11,12]. This was associated with an inability to appropriately increase β-cell proliferation. Thus, exposure to a LP diet in utero is associated with mice being unable to adequately regenerate β-cell mass after injury, to maintain β-cell viability in the presence of cytotoxic cytokines, and to increase β-cell mass to compensate for the metabolic demands of pregnancy.

Hormonally-driven insulin resistance is an important feature of the metabolic adaptation to pregnancy. This is compensated by the pregnancy-associated increase in β-cell mass and glucose-stimulated insulin release to maintain maternal euglycemia [13]. Van Assche et al. [14] reported a doubling of fractional β-cell area in women who died in third trimester or at parturition, when compared to age-matched, non-pregnant subjects. A similar study by Butler et al. [15] found a 1.4-fold increase β-cell area but included women who died in first trimester before the maximum increase in β-cell mass would be attained. Importantly, an implied failure of β-cells to undergo adaptive change after first trimester has been linked with gestational diabetes mellitus (GDM) [16].

The cellular mechanisms by which the adaptability of β-cell mass becomes compromised following exposure to LP diet in early life includes changes in the expression of peptide growth factors, such as insulin-like growth factor 1 (IGF1), and in Reg genes [17]. Adult offspring of LP-fed mice also show changes in signaling through the mammalian target of rapamycin (mTOR) pathway in pancreatic islets [18]. mTOR is a widely expressed serine/threonine kinase that links nutritional and environmental signals with those initiated by growth factors and hormones, resulting in a coordination of cell proliferation, cell hypertrophy, protein translation and metabolism [19,20,21]. mTOR exists in two functional complexes, mTOR Complex 1 (mTORC1) and mTORC2, with Raptor being one of a number of adaptor proteins contributing to mTORC1, and Rictor being an adaptor protein within mTORC2. mTORC1 phosphorylates ribosomal p70S6 kinase1 (p70S6k1) and eukaryote initiation factor 4E-binding protein1 (4E–BP1), causing increased protein translation including that of insulin, whereas mTORC2 phosphorylates PKCα and Serum and Glucocorticoid-regulated Kinase 1 (SGK1) [22,23]. Amino acids and growth factors activate mTORC 1 signaling through the activation of phospho-inositol 3 kinase (PI3K) and Akt, resulting in the activation of Ras homolog enriched in brain (Rheb) GTPases. Rapamycin is a specific mTORC 1 inhibitor, although it may also inhibit mTORC2 after prolonged incubation at high doses [24,25]. The integration of up-stream signals controlling mTORC activity is dependent on the tuberous sclerosis complex (TSC) genes [26].

Rapamycin inhibits β-cell proliferation in vitro [27,28], and prevents the expansion of β-cell mass during pregnancy in the mouse [29], acting through inhibition of mTORC1 [30]. Rapamycin also prevents adaptation of the pancreatic β-cell mass to hyperglycemia [31]. Conversely, activation of mTOR signaling by conditional deletion of *TSC2* in β-cells resulted in cell proliferation and hypertrophy [32,33]. Growth factors such as IGF1 enhance β-cell survival through anti-apoptotic pathways mediated by Akt, whose actions are mediated by mTOR signaling [34]. Hence, mTORC1 signaling is likely to be central to the control of β-cell mass and plasticity through altering cell cycle kinetics and proliferation, by promoting β-cell survival, and maintaining insulin release through the control of protein translation. In accordance with this concept, the administration of a LP diet to young rats caused a decrease in the islet content of mTOR protein and glucose and amino acid-stimulated insulin release [35]. In islets from offspring of LP-fed rats, decreased nutrient-stimulated insulin release was accompanied by a decreased activity of the mTORC1 target, ribosomal protein S6 kinase β-1 (S6K1) [36].

Signaling through the mTOR pathway is also present in pancreatic α-cells and long-term changes in glucagon secretion following exposure of mice to LP diet in utero could also contribute to glucose intolerance during pregnancy. Targeted deletion of Raptor in α-cells in mice to functionally disable mTORC1 showed mTOR signaling to be important for the functional maturation of α-cells around the time of weaning [37]. Alpha-cell mass became deficient with increasing age, associated with decreased glucagon content, and release in response to hypoglycemia. Additionally, the ability of insulin to increase α-cell proliferation in the α-TC1 cell line is mediated by mTOR signaling [38]. Administration of LP diet to post-weaning mice resulted in an increase in α-cell mass and a decreased ability of glucose to down-regulate glucagon secretion [39]. An elevated glucagon secretion was reported previously by us at 130 days age in rats exposed to LP diet in utero [40], but it is not known if this becomes established in early life.

Exposure to LP diet in early life also causes long-term changes to the innate immune system in the offspring, including inflammasome gene expression, macrophage function, and the ability to combat bacterial infections [41,42,43]. The capacity of tissues to mount an immune response to infection involves the stimulator of interferon (IFN) genes (STING) (also known as *TMEM173*) signaling pathway since STING recruits TANK-binding kinase 1 (TBK1), which is able to activate interferon regulatory factor 3 (IRF3) leading to its translocation to the nucleus where it increases the transcription of type I IFNs. An inflammatory environment, as exists in the mother during nutritional deficiency [44] suppresses mTORC1 activity and dysregulates cellular metabolism [45]. The formation of a p70S6k-STING-TBK1 complex downstream of mTORC1 [46] suggests that long-term changes in mTOR signaling in the islets of LP-exposed offspring may not only alter pancreatic endocrine function but also the capability of the innate immune system. Evidence suggesting the involvement of STING in metabolic pathways is recent, but its pivotal role in inflammation, DNA damage responses, and triggering of anti-viral defenses against DNA and RNA viruses is well documented [47]. The presence and cell localization of STING within islets and how this might alter in offspring of LP-fed mice has not previously been reported.

The purpose of this study was to examine changes in the expression and function of proteins involved in mTOR signaling in islets from mice exposed to LP vs. control diet in utero, to determine if prenatal programming of the pancreatic islet mTOR axis might contribute to the longer-term phenotype of decreased β-cell mass, function and adaptability to metabolic stress that could contribute to gestational diabetes. Thus, we have concentrated our studies on possible changes in mTOR signaling early in postnatal life prior to and after weaning (days 7 and 30) as a result of the altered nutritional environment in utero. The long-lasting effects of such changes to mTOR activity were studied at 130 days age when rats exposed to an LP diet in utero exhibit abnormal glucose tolerance [8].

## 2. Materials and Methods

### 2.1. Animals 

Balb/c mice (Charles River Ltd, Montreal, QC, Canada) were housed in a temperature-controlled room in a 12-h light:dark cycle. Female mice were mated, and pregnancy was confirmed by a vaginal plug. Pregnant mice were randomly assigned to a control (C; 20% protein) or a low-protein (LP; 8% protein) diet similar to that described by Snoeck et al. [48], who also demonstrated no change in palatability of the diets through measurement of daily food consumption. The two diets were isocalorific, the deficiency in calories in the LP diet being compensated by additional carbohydrate (Supplementary Table S1). Food and water were given ad libitum. All mice were transferred to the control diet following parturition. Body weight was recorded prior to sacrifice by CO_2_ asphyxia after an overnight fast, blood taken for glucose determination using a glucose meter (Accu-Check, Roche, Diagnostics, Laval, QC, Canada), and the pancreas removed at postnatal day 1,7,30, or 130 and weighed. Approximately equal numbers of male and female offspring were included in the study. All animal procedures were approved by the Animal Care Committee of the University of Western Ontario (Approval number: 2018–027) in accordance with the guidelines of the Canadian Council for Animal Care.

### 2.2. Immunohistochemical and Immunofluorescence Staining

Pancreata or isolated islets were fixed in 4% paraformaldehyde, embedded in paraffin, and three 5 μm tissue slices prepared along the longitudinal axis of the pancreas with separation of at least 150 μm. Antigen retrieval was performed using 10 mM sodium citrate with 0.05% (*v/v*) Tween 20. Tissues were washed with phosphate buffered saline (PBS) followed by a wash in Tris-buffered saline with 0.05% (*v/v*) Tween 20 (TBST). Sniper (Biocare Medical, Pacheco, CA, USA) was used to block non-specific protein binding. Dual immunofluorescent immunohistochemistry was performed to co-localize either mTOR and insulin, mTOR and glucagon, or insulin and EdU (nuclear antigen of DNA replication), as described by Beamish et al. [49]. Tissues were incubated overnight with rabbit polyclonal anti-mTOR primary antibody (1:300; Abcam, Cambridge, MA, USA), rabbit polyclonal anti-insulin (1:200; Santa Cruz Technologies, Santa Cruz, TX, USA), or rabbit polyclonal anti-glucagon (1:200, glucagon anti-rabbit, Santa Cruz) as required at 4 °C. All antisera were diluted in Antibody Diluents (Dako, Agilent Technologies, Santa Clara, CA, USA) with 0.05% (*v/v*) Tween 20. The following day, secondary antibodies (1:500 Invitrogen, Eugene, OR, USA) were applied against the primary antibody using 555 and 488 fluorophores, respectively. After a 2 h incubation at room temperature in the dark, DAPI (4,6-diamidino-2 phenylindole), dihydrochloride, (1:500, Thermo Fisher Scientific, Waltham, MA, USA) was applied for 5 min to counterstain nuclei in the case of isolated islets. Coverslips were mounted with fluorescent mounting medium and stored at 4 °C (Dako, Agilent). Three sections from each pancreas were analyzed for immunofluorescence under a Zeiss fluorescence Axioskop microscope (Carl Zeiss, Oberkochen, Germany). To detect apoptosis, an In Situ Cell Death Detection Kit (Roche Applied Science, Penzberg, Germany) with a terminal deoxynucleotidyl transferase (TdT) dUTP Nick-End Labeling (TUNEL) assay was used following staining for insulin to identify β-cells. The presence of EdU was detected using the Click-iT EdU Imaging Kit (Thermo Fisher).

For the visualization of mTOR, pancreas sections were also examined using a diaminobenzidine (DAB) chromogen. Following prior exposure to 3% (*v/v*) H_2_O_2_ to block endogenous peroxidase activity followed by exposure to primary antibodies for mTOR or insulin, tissues were then incubated with biotinylated goat anti-rabbit IgG secondary antibody (1:200; Vector Laboratories, Burlingame, CA, USA) for 2 h at room temperature followed by a 2 h incubation with ExtrAvidin Peroxidase (1:100; Sigma-Aldrich, St. Louis, MO, USA) at room temperature. mTOR was visualized using Liquid DAB Substrate (BioGenex, San Ramon, CA, USA), and tissues were counterstained with Carrazi’s hematoxylin. Dehydrated sections were mounted under glass coverslips with Permount (Fisher Scientific Toronto, ON, Canada). The cellular area of insulin-immunoreactive cells co-staining for mTOR for least 20 islets from each pancreas was calculated using Image J software (NIH). Beta-cell mass were calculated from 3 sections per pancreas as described by Chamson-Reig et al. [50]. Total pancreas sectional area was imaged with Northern Eclipse software (EMPIX Imagining Inc., Mississauga, ON, Canada) using brightfield microscopy at 2.5X magnification and the area traced using ImageJ. All insulin (β-cells)-positive cells were imaged (Nikon Eclipse Ts2R, Nikon, Minato, Tokyo, Japan) at 20X magnification using NIS elements software (Nikon). Beta-cell area was traced manually using ImageJ software and β-cell mass calculated by dividing total insulin-positive cell area by total pancreas sectional area, then by multiplying by pancreas weight.

Mouse and human pancreata were examined for the presence of STING using fluorescent immunohistochemistry. Formalin-fixed, paraffin embedded sections of human pancreas from the Exeter Archival Diabetes Biobank (EADB; http://foulis.vub.ac.be/) were used. These comprised samples from non-diabetic patients (aged 4 and 80 years, both female). Human tissues were studied with full ethical approval and following the adequate reporting standards for human studies. After standard dewaxing and rehydration, samples were subjected to heat-induced epitope retrieval (HIER) in Citrate pH6 then probed in a sequential manner with STING (Abcam), Glucagon (Cell Signaling, Beverly, MA, USA), and Insulin (Dako, Agilent). The relevant antigen-antibody complexes were detected using secondary antibodies conjugated with fluorescent dyes (Alexa Fluor anti-mouse 488 TSA, anti-rabbit 555, anti-guinea pig 647) (Invitrogen, Paisley, UK). Cell nuclei were stained with DAPI. After mounting, images were captured with a Leica AF6000 microscope (Leica, Milton Keynes, UK) and processed using the standard LASX Leica software platform. 

### 2.3. Cell Lines

The mouse pancreatic alpha cells (αTC1-6) were a gift from Dr. S. Dhanvantari, Lawson Health Research Institute, originally purchased from ATCC (Rockville, MD, USA) and grown in Dulbecco’s Modified Eagle’s Medium (DMEM) (Invitrogen, Burlington, ON, Canada) supplemented with 15% (*v/v*) horse serum, 2.5% (*v/v*) fetal calf serum, 25 mM D-glucose in the presence of penicillin and streptomycin. Cells were cultured in a humidified incubator at 37 °C and 5% CO_2_ and passaged to new flasks when confluent. The mouse MIN6 β-cells were a gift from Dr. Dawn Kilkenny, University of Toronto, and grown in DMEM supplemented with 15% (*v/v*) fetal calf serum, 25 mM D-glucose, 50µM beta mercaptoethanol, penicillin and streptomycin.

### 2.4. Islet Isolation

After removal pancreata were perfused with 1 ml of 1mg / ml Collagenase type V (Sigma-Aldrich, St. Louis, MO, USA) in Hank’s Buffered Salt Solution (HBSS; Invitrogen). Samples were collected in tubes containing 4 ml of 1 mg / ml Collagenase type V and placed in a shaking water bath (37 °C) for up to 25 min. Following tissue digestion tissue was passed through a 14-gauge needle, washed in HBSS containing 5% (*w/v*) bovine serum (Invitrogen), and islets separated using a modified sequential Dextran gradient protocol [51]. Islets were picked by hand using an inverted microscope and incubated overnight at 37 **°**C in RPMI media (Sigma-Aldrich). Islets were the re-picked and incubated in Roswell Park Memorial Medium (RPMI) containing 6.5 mM D-glucose, 10% (*v/v*) fetal calf serum, and 1% Penicillin/Streptomycin for up to 72h in the presence or absence of rapamycin (Sigma-Aldrich) solubilized in dimethyl sulphoxide. Alternatively, islets were centrifuged at 1000 RPM for 5 min and pellets frozen at −80 °C for subsequent RNA or protein extraction.

### 2.5. Western Blot Analysis

For Western blot analysis using isolated islets, proteins were extracted using standard Tris lysis buffer containing protease inhibitors and were sonicated and centrifuged at 13,000 RPM for 15 min at 4 °C. Supernatants were removed and proteins were quantified using a Micro BCA Protein Assay Kit (Thermo Fisher Scientific). Twenty micrograms of total islet protein were loaded onto a NuPAGE Novex Bis-Tris 4–12% gel and electrophoresed at 200 V for 35 min. The proteins were transferred to a PVDF membrane using an iBlot Gel Transfer Device (Invitrogen) and blocked with 5% (*w/v*) skim milk in TBST for 1 h at room temperature. Membranes were then cut into horizontal strips based on the expected molecular weight position for mTOR (289 kDa), Raptor (150 kDa), Akt (60 kDa), p70S6k (70 and 85 kDa) and β-actin (41 kDa) relative to a standardized protein molecular weight ladder, and each strip was incubated with the appropriate primary antibody. These were either monoclonal anti-mTOR primary antibody (1:500), monoclonal anti-Akt (1:250), rabbit polyclonal anti-p70S6K (1:500), or monoclonal anti-Raptor (1:500) overnight at 4 °C (mTOR and Raptor antibodies left for 48h). All the above antibodies were purchased from Cell Signaling. Membranes were then incubated with horseradish peroxidase-labelled goat anti-rabbit IgG antibody (1:10,000, Abcam) for 2 h at room temperature. Bands were visualized using SuperSignal West Pico Chemiluminescent Substrate (Thermo Fisher Scientific) and quantified by densitometry using Image Lab software (Bio-Rad). For visualization of phosphorylated mTOR, Akt and p70S6k the membranes were stripped using antibody stripping buffer (Gene Bio-Application, Israel) and vigorous washing in distilled water. Membranes were blocked with 5% (*w/v*) BSA in TBST for 2h at room temperature, then re-incubated with polyclonal anti-phosphorylated Seri2481 mTOR (1:250), monoclonal anti-phosphorylated Akt (1:1000) or polyclonal anti-Thr389 phosphorylated p70S6K (1:250), respectively prior to visualization and quantification of the corresponding phosphorylated proteins (all antibodies from Cell Signaling). β-actin was used as a comparative control for all analyses and was visualized using a 1:10,000 dilution polyclonal antibody (Abcam).

### 2.6. Quantitative Real-Time Polymerase Chain Reaction (qRT-PCR)

Total RNA was extracted from isolated islets from 7 or 30 day old mice using an RNeasy Plus Mini kit (Qiagen, Toronto, ON, Canada). The quality of the RNA was examined using the Agilent 2100 Bioanalyzer (Agilent Technologies Inc., Mississauga, ON, Canada) and RNA 6000 Nano kit (Caliper Life Sciences, Hopkinton, MA, USA). Any DNA contamination was removed from the samples using DNase (Invitrogen). RNA was reverse transcribed using iScript Reverse Transcription Supermix (Bio-Rad Laboratories, 170–8840). Quantitative polymerase chain reaction (PCR) experiments were accomplished using the 2^ΔΔC^_T_ method after confirmation of parallel PCR amplification efficiencies on a Real-Time PCR ABI Prism 7500. IQ SYBR Green (Bio-Rad, 170–8882) or TaqMan Fast Advanced Master Mix (Applied Biosystems, Thermo Fisher Scientific) were employed for detection of PCR products, with primer sequences and sources provided in Supplementary Table S2. The TaqMan qPCR was performed on triplicate samples with an initial polymerase activation step at 95 °C for 20 s, followed by 40 cycles of denaturation (95 °C for 3 s) and annealing/extension (60 °C for 30 s). PCR reactions for TSC2 and proglucagon using non-TaqMan primers were performed using an initial denaturation at 95 °C for 5 min, followed by cycles of denaturation (95 °C for 15 s), primer annealing (60 °C for 1 min) and transcript extension (50 °C for 2 min) for 40 cycles. TaqMan primers were purchased from Applied Biosystems and SYBR Green primers for TSC2 and proglucagon were purchased from Sigma-Aldrich. These primers were designed using National Center for Biotechnology Information (NCBI) Primer Blast, National Center for Biotechnology Information, Bethesda, MD, USA.

### 2.7. Statistical Analysis

Data are presented as mean ± standard deviation (SD) or standard error of the mean (SEM) with a significance level of *p* < 0.05. Analysis of variance (ANOVA) was used to determine significant differences resulting from diet followed by a Bonferroni post-hoc test or an unpaired *t* test. Examination of the variance between the approximately equal numbers of males and female mice in the present study showed no significant differences between the sexes for any measured parameter at any time point and they were therefore combined for analyses.

## 3. Results

**Figure 1:**
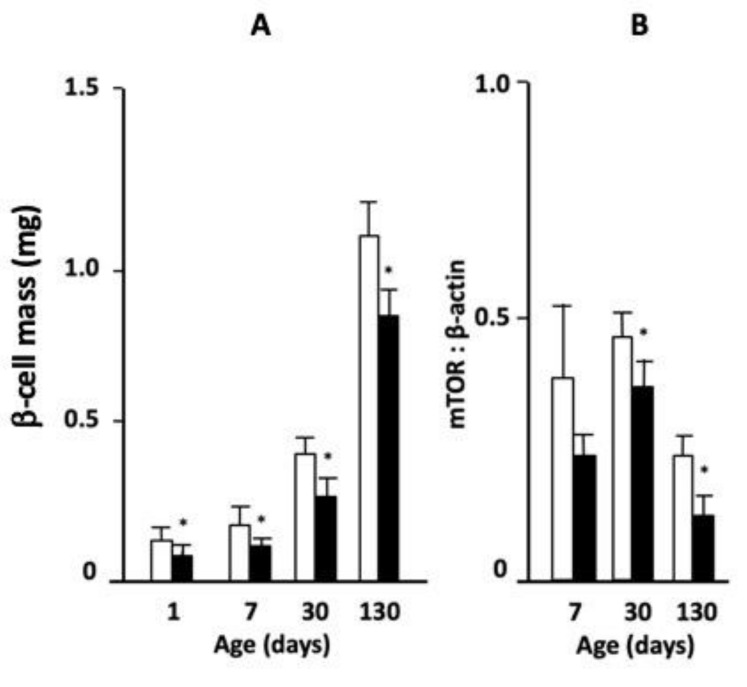
(**A**) Changes in β-cell mass in offspring from control (open bars) or low-protein (LP) diet (closed bars)-fed mice at 1, 7, 30 or 130 days of age, and (**B**) abundance of mammalian target of rapamycin (mTOR) protein in isolated islets relative to β-actin at 7, 30 and 130 days (Mean ± standard error of the mean (SEM); * *p* < 0.05 vs. control, *n* = 6).

Mice born to mothers who had received LP or control diet during gestation were followed from the day of birth until fully grown at 130 days of age. Body weight was significantly lower in LP diet-exposed animals at days 1 and 7 but did not differ from that of control diet-exposed mice by days 30 and 130 (Table 1). Pancreas weight as a percent of body weight was reduced in LP-fed offspring at day 7, but not at other ages. However, β-cell mass was significantly lower in offspring from LP-fed mothers compared to control diet throughout postnatal life (Figure 1A). Despite the reduction in β-cell mass in the LP-exposed offspring, fasting blood glucose did not differ from control-fed animals at any age (Table 1). The abundance of mTOR protein in isolated islets relative to β-actin, as determined by Western blot, was significantly lower in the mice exposed to LP diet in utero than control-fed animals at 30 and 130 days of age (Figure 1B, representative images of Western blots are shown in Supplementary Figure S1). Thus, while exposure to LP diet in early life had no long-lasting effects on body or pancreas weight there were long-term deficits in β-cell mass and pancreatic mTOR presence.

**Figure 2:**
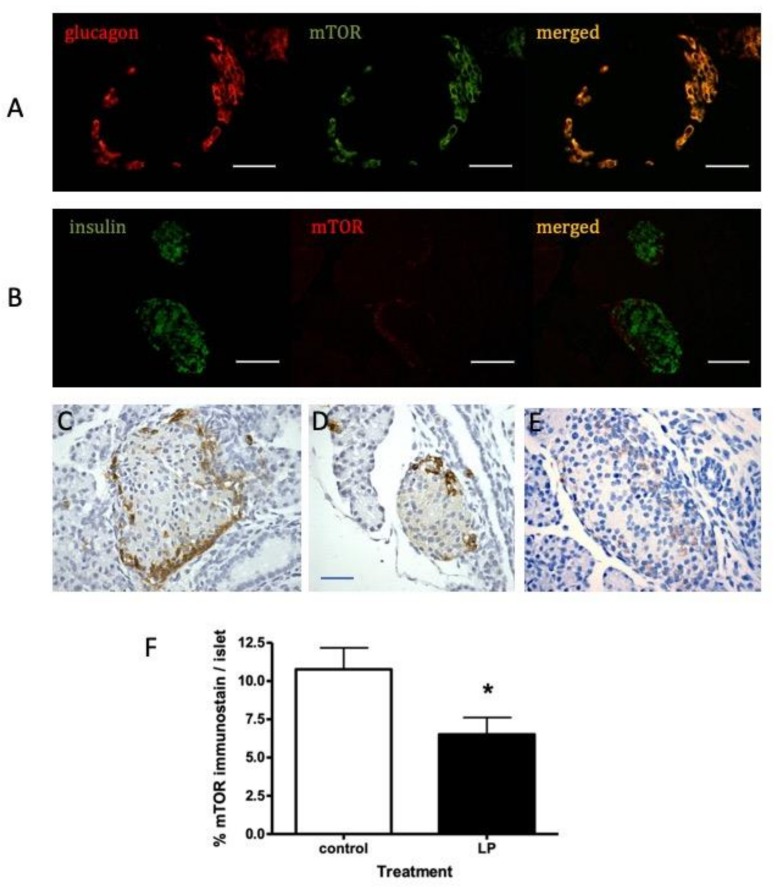
Representative images of immunofluorescent staining showing co-localization of (**A**) glucagon (red) and mTOR (green) and (**B**) insulin (green) and mTOR (red) in pancreatic islets from control-diet fed mouse at age 30 days (6 mice examined). Immunohistochemical localization of mTOR in similar islets is shown using diaminonezidine (DAB) chromogen in islets from 7 day old control diet-fed mice (**C,D**) or LP diet (**E**). The percentage area of islets occupied by mTOR-positive cells is shown for control or LP diet-fed mice in panel **F** (mean ± SEM; *n* = 6, * *p* < 0.05 vs. control diet). Scale bars represents 50 µm.

To determine the cellular location of mTOR within the mouse pancreas, tissues were examined firstly by immunofluorescence. Co-staining for mTOR and either insulin or glucagon showed that mTOR was co-located within the α-cells of islets at 30 days of age (Figure 2A,B). To provide higher cellular resolution, these studies were repeated using immunohistochemistry for islets from 7 day old mice (Figure 2C,E). MTOR presence was strongest in the rim of the islets predominantly occupied by α-cells, although a weaker stain was visible in the β-cell-rich core of the islets. A similar distribution was seen at 130 days (Supplementary Figure S2). Compared to islets in pancreata from offspring exposed to control diet in utero, a less intense mTOR staining was apparent in islets from animals exposed to LP diet (Figure 2E). Comparison of the mean islet area occupied by mTOR showed this to be significantly reduced in islets from LP diet-exposed mice (Figure 2F). To confirm that mTOR presence in mouse α- as well as β-cells was sensitive to rapamycin the αTC1-6 α-cell line and MIN6 mouse β-cell line were exposed to rapamycin to determine the changes in phosphorylated (p) mTOR presence. Activated mTOR was detectable in both cell lines and was significantly decreased by rapamycin in both (Supplementary Figure S3).

Similarly, we sought confirmation that the mTOR signaling pathway was functionally relevant to islet cell proliferation and survival in young mice using isolated islets from control diet-fed animals of 30 days age. Cell DNA replication was visualized by the localization of EdU in histologically fixed islets co-stained for insulin (Supplementary Figure S4A) while cellular apoptosis was visualized using TUNEL immunohistochemistry (Supplementary Figure S4C), with and without incubation with rapamycin. Islet DNA synthesis was significantly prevented by rapamycin (Supplementary Figure S4B) while apoptosis was significantly increased (Supplementary Figure S4D). This confirmed that the mTOR present in young mice islets could contribute towards DNA synthesis and cell survival in both β- and non-β-cells.

**Figure 3:**
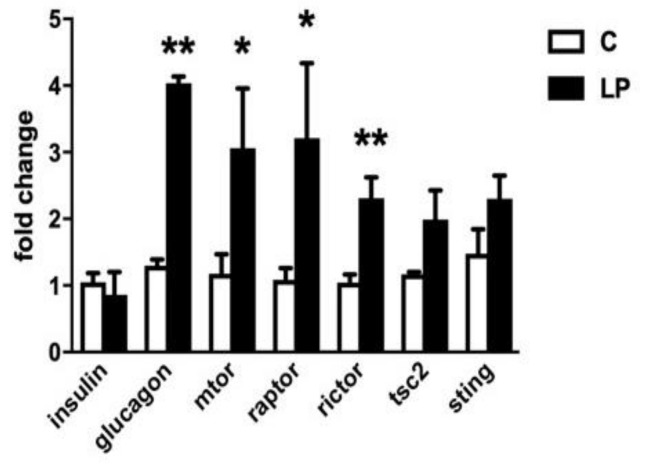
Fold changes relative to the expression of glyceraldehyde-3-phosphate dehydrogenase (GAPDH) in the expression of mRNA encoding insulin, proglucagon, mTOR, Raptor, Rictor, TSC2 or STING within isolated islets from pancreata of 7-day old mice exposed in utero to control (C, open bars) or LP (closed bars) diet. Values show mean ± SEM; *n* = 3–7 samples from different animals per group, * *p* < 0.05, ** *p* < 0.005 vs. control diet.

To further examine changes in the mTOR signaling pathway in mice born to LP diet-fed mothers mRNA levels were quantified in isolated islets from 7-day old animals for mTOR, Raptor, Rictor and TSC2 compared to islets from control diet-exposed mice (Figure 3). The levels of mRNA for mTOR, Raptor and Rictor in islets were all significantly higher in mice exposed to LP diet, while TSC2 expression was unaltered. In the same islets insulin expression did not differ with previous diet, but the level of proglucagon gene expression was increased 3.2 ± 0.2-fold in LP diet-exposed mice. Proglucagon gene expression was similarly significantly increased in islets from LP-exposed animals at 30 days age vs. control diet (2.15 ± 0.6, *p* < 0.01, n = 6). We also measured the expression level of STING, a protein expressed by β-cells as part of the innate immune system defence against cellular stress including infection with pathogenic or damaged double-stranded DNA. STING expression in islets was not found to differ with prior exposure to control of the LP diet.

**Figure 4:**
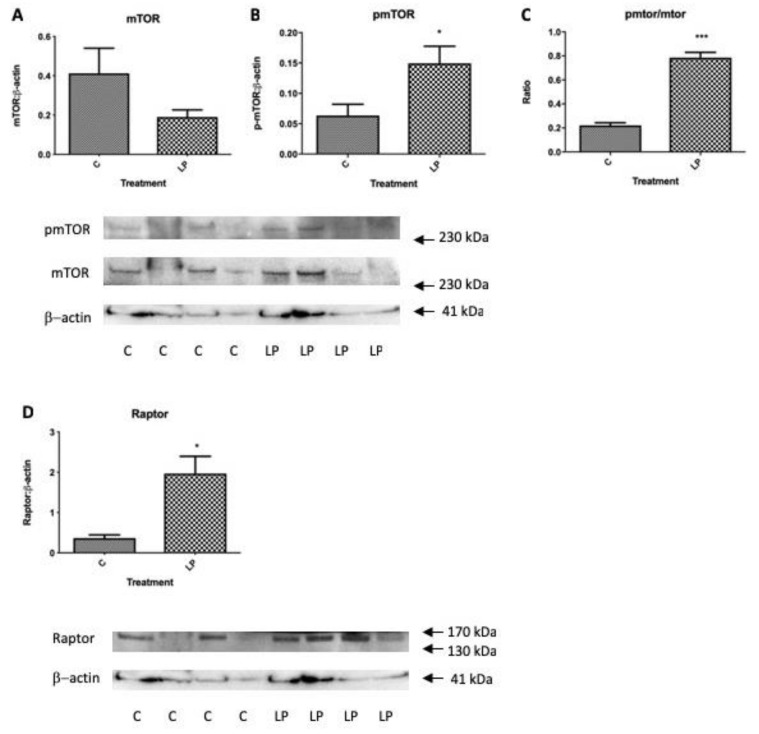
Changes in the abundance of total mTOR protein, phosphorylated mTOR and their ratio (**A**–**C**), and Raptor (**D**) in isolated islets from pancreata of 7-day old mice exposed in utero to control or LP diet relative to β-actin. Values show mean ± SEM; *n* = 4 per group, * *p* < 0.05, *** *p* < 0.001 vs. control diet. Confirmation of molecular weight was obtained relative to a standard protein molecular weight ladder (closest standards indicated by arrows).

**Figure 5:**
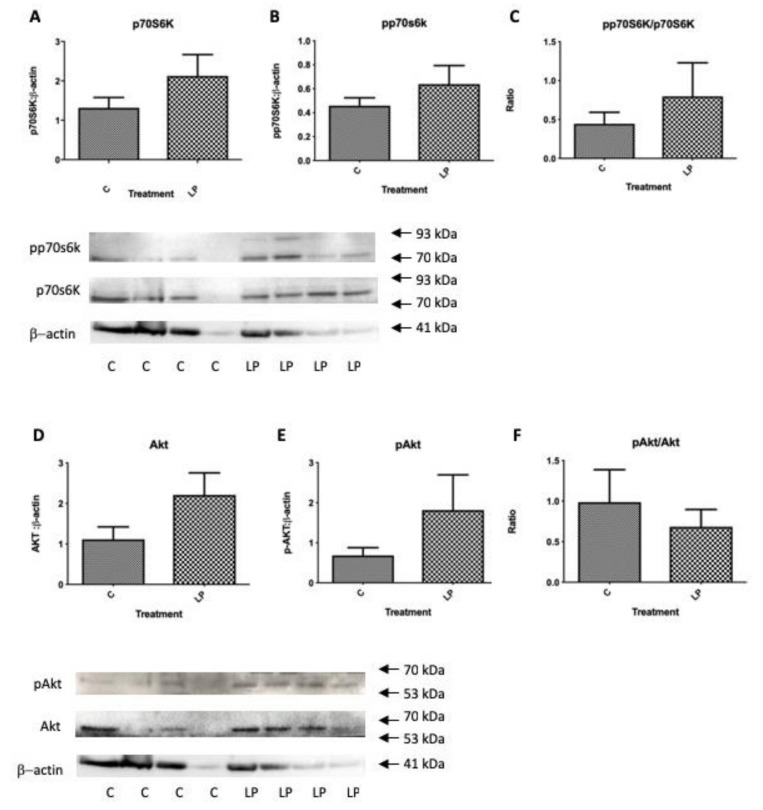
Changes in the abundance of total p70S6k, phosphorylated p70S6k and their ratio (**A**–**C**); and total Akt, phosphorylated Akt and their ratio (**D**–**F**) in isolated islets from pancreata of 7-day old mice exposed in utero to control or LP diet relative to β-actin. Values show Mean ± SEM; *n* = 4 per group, Confirmation of molecular weight was obtained relative to a standard protein molecular weight ladder (closest standards indicated by arrows).

Within the same isolated islet pools used to determine gene expression levels in Figure 3, the levels of total and phosphorylated mTOR were determined together with the levels of key up- and down-stream genes influencing mTOR actions on protein synthesis and cell proliferation, Akt and p70S6k. Protein levels of Raptor that contributes to the mTORC1 complex was also determined. Levels of total islet mTOR tended to be lower in islets from LP diet exposed mice in agreement with Figure 1B, although the proportion of active, phosphorylated mTOR was significantly greater as was the ratio of active to total mTOR (Figure 4A–C). The level of Raptor was also significantly higher in mice exposed to LP diet (Figure 4D). Despite the altered presence of mTORC1 components there was no significant difference in total, activated or the ratio between the two of p70S6k or of Akt (Figure 5A–F). Thus, although islets from young mice exposed to LP diet showed changes in mTORC1 complex that would be expected to favour increased protein synthesis and cell proliferation, these were not accompanied by a significantly altered activation of p70S6k or Akt, or altered insulin gene expression (Figure 3), although pro-glucagon gene expression was increased.

**Figure 6:**
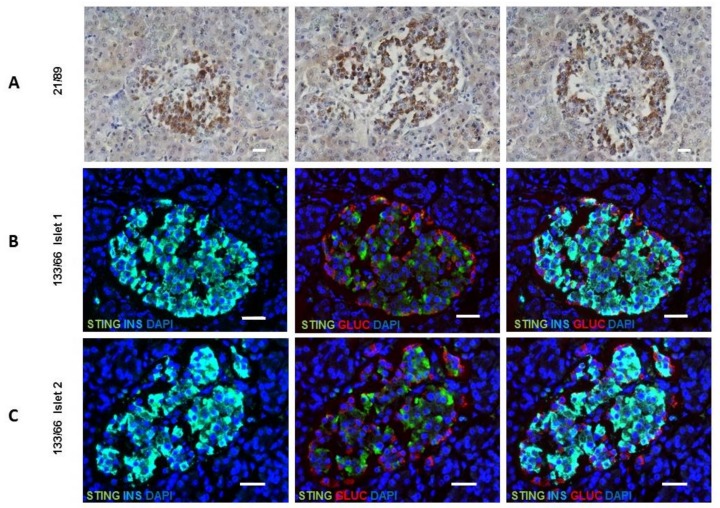
STING expression is present in the islets of human individuals in both early and late life. (**A**) Immunohistochemical localization of STING in 3 different islets from an individual (sample number 21/89) aged 4 years using DAB chromogen. (**B**,**C**) Images demonstrating that STING is localized to the β-cell. Immunostaining for STING (green) and insulin (light blue; left panel), STING (green) and glucagon (red; middle panel) or STING (green) glucagon (red) and insulin (light blue; right panel) is shown in two representative islets from an older individual (sample number 133/66) aged 80 years. Scale bars represent 30 μm.

Finally, the cellular localization of STING was examined in both mouse and human pancreas since this has not been previously described for any species. We assessed the presence of STING in mouse pancreas by immunohistochemistry at 30 days age and found STING to largely co-localize with β-cells within the islet core (Supplementary Figure S5). When pancreas sections were studied from people without diabetes STING immunostaining was restricted to the islets and present mainly within the insulin-containing β-cells (Figure 6). STING was abundant in all samples and demonstrated using both immunohistochemistry (sample number 21/89: aged 4 years) (Figure 6A) and immunofluorescence (sample number 133/66: aged 80 years) (Figure 6B,C). The role of STING in anti-viral defense, DNA damage response, and metabolism is unknown in the context of diabetes.

## 4. Discussion

The model of LP diet administration during mouse pregnancy significantly reduced the weight of offspring at birth but by 30 days age no body weight differences were found between mice previously exposed to control or LP diet. Similarly, while the relative weight of the pancreas was reduced a week following birth in LP diet-fed offspring, no differences were seen at day 30 or subsequently. We previously reported that adiposity was also not increased in LP-exposed offspring up to 130 days age [10], which differs from the increased tendency for small for gestational age human infants to exhibit increased obesity as adults [52]. Despite the absence of adult obesity in mice who received LP diet, the β-cell mass was significantly reduced from birth until adulthood, suggesting that this resulted from intrinsic deficiencies in β-cell generation rather than any secondary detrimental effect of adipose-derived adipokines, such as leptin, in adulthood [53]. We reported previously that pancreas from 30-day-old mice receiving LP diet in utero had significantly reduced expression of growth factors and transcription factors necessary for β-cell generation and proliferation, such as IGF-1 and pancreatic and duodenal homeobox 1 (Pdx1) [17]. Also, in a previous study [10] we found small differences in β-cell mass between male and female mice at day 30 with respect to prior administration of LP diet in utero and no difference in β-cell mass at 130 days between LP and control groups. In the present study we found no significant differences between the sexes with respect to β-cell mass at any time point, and both sexes were, therefore, combined for analysis. Using a greater sample size we now report a lower β-cell mass in the LP-exposed animals at all time points compared to a control diet.

Although a reduction in islet mTOR presence might have been anticipated in offspring experiencing a reduced nutrient environment prior to birth and exhibiting a reduced β-cell mass, this was not so by 7 days of age but was present by 30 days, when the area of islet cells staining for mTOR presence was also reduced, and subsequently. However, mTOR mRNA expression and the proportion of mTOR that was phosphorylated, and therefore active, was significantly increased at 7 days of age. Similarly, the mRNA levels and protein presence for Raptor were significantly greater in pancreas from LP-fed offspring. However, the level of expression of insulin was no different between animals that received LP or control diet. These findings may be explained by total islet mTORC activity representing the complex net effects of mTOR transcription and translation, co-complex protein expression such as Raptor, and inhibitors of complex activation such as TSC2 in both the α- and β-cells. The observed distribution of mTOR protein was more strongly associated with α-cells than β-cells from at least day 7 until age 130 days. We and others reported previously using the same model that α-cell mass was also increased in LP-exposed mice at 14 and 30 days but was no different from control-fed animals by adulthood [10,54]. In agreement with a relative hyperplasia of α-cells in early postnatal life we here found that proglucagon gene expression was also relatively elevated. Thus, the increased expression of mTOR, Raptor and Rictor, accompanied by a higher proportion of active mTOR protein, in islets from LP-exposed mice may reflect predominantly altered mTORC signaling within the α-cell population rather than the β-cells. However, mTOR is present and active in both cell types, as demonstrated here by the inhibition of mTOR with rapamycin in both α- and β-mouse cell lines, and the ability of rapamycin to inhibit cell proliferation in isolated mouse islets while increasing islet cell apoptosis in both insulin and non-insulin containing cells. An increased presence of activated mTOR and Raptor in islets from LP-exposed mice at age 7 days might be expected to reflect an altered upstream activation of Akt or downstream activation of p70S6k, but this was not seen. It is likely that if the majority of mTORC signaling represented activity within the α-cells and that any associated changes in Akt or p70Sk signaling were diluted by the presence of the more numerous β-cells.

A long-lasting re-distribution of cell mass and hormonal release and actions between α- and β-cells in response to LP diet in utero may well be precipitated by changes in mTOR signaling. When LP diet was administered in utero to rats, glucagon release in response to amino acids was still enhanced in adulthood [40]. These observations are supported by the finding that LP food restriction postnatally in mice also increases α-cell mass while diminishing the ability of glucose to reduce glucagon secretion [39]. Hepatic glucagon sensitivity was also reduced. While the impact of LP diet postnatally on glucagon secretion is reversible, exposure to LP diet in utero appears to have a longer-term impact, consistent with fetal tissue re-programming.

Several mechanisms may contribute to the ability of a LP diet environment in utero to re-program mTOR signaling pathways in either α- and β-cells, including an elevated pro-inflammatory environment, levels of cellular stress, changes in the levels of islet cell miRNAs, or changes in islet cell lineage development. Many of these mechanisms have been linked to altered signaling through mTORC complexes. Rat pups born to mothers receiving LP diet during gestation demonstrated increased serum and hepatic levels of IL-6 and TNF-α at weaning [55] compared to control-fed animals, and similar results were found for the expression of IL-6 in adipose tissue macrophages [56]. Reports on the expression of the IL-6 receptor in the pancreas are conflicting but suggest IL-6 is found in both the α- and β-cells. Within the β-cells IL-6 activates STAT3 and potentiates pro-inflammatory cytokine-induced cell death [57]. Conversely, Ellingsgaard et al. [58] showed that IL-6 stimulates α-cell proliferation while preventing apoptosis. With regards to TNF-α, it can act through Akt to enhance NF-kappaB (NFkB) activity, a transcription factor involved with cell replication, in a pathway that is dependent on the presence of Raptor and the inactivation of TSC1/2 complex [59,60]. One contrary study found that inhibition of mTORC1 is necessary for IL-6 to protect β-cells from apoptosis through the inhibition of autophagy [61]. The actions of mTOR are altered when cells are exposed to stress conditions such as the generation of reactive oxygen species as occurs during dietary-induced fetal growth retardation [3,62]. This is mediated through modulation of the TSC1/2 complex and its ability to inhibit mTORC signaling.

There is accumulating evidence that miRNAs can alter the developmental patterns of pancreatic endocrine cells, and that these are susceptible to environmental influences in utero. The suppression of β-cell proliferation in offspring of mice exposed to LP diet in utero is mediated in part by an increased presence of MiR-15b which inhibits cyclin D1 and D2 activity [63]. A similar action of MiR375 and several other MiRs was reported in β-cells from rats exposed to LP diet in utero [64]. Of these, neutralization of MiR199 or 342 using antibodies resulted in an increased abundance of mTOR protein and increased insulin release from dispersed mouse β-cells from animals previously exposed to LP diet. Such changes in MiRNAs need to be considered in the context of an altered trajectory of islet endocrine cell development in utero when exposed to a LP environment. Islets from offspring of rats whose mothers received LP diet throughout gestation showed an increased expression of transcription factors such as Pdx1, hepatocyte nuclear factor 1 alpha (Hnf1α) and Hnf4α at weaning, indicative of premature functional maturation of the β-cells at the expense of a reduced β-cell mass [65].

Here, we first report that STING is abundantly expressed within the insulin-containing β-cells of mouse and human islets and is present in both early and late life. Since p70S6k can complex with STING, resulting in activation of IFN production [46], we anticipated that an alteration in islet mTOR activation in mice previously exposed to LP diet might result in a similarly altered p70S6k activity and a corresponding change in STING expression. However, within total islet protein the abundance and activation of p70S6k did not change after LP diet. Moreover, while mTOR was predominantly visualized within α-cells, STING is mainly localized to β-cells. While this in intriguing the metabolic, inflammatory and anti-viral functions of STING within the β-cell remain unknown in both healthy individuals and individuals with a disease of the pancreas. STING is likely to be operative is islets throughout life and further studies would elucidate any roles in inflammation or metabolic deregulation within gestational diabetes and type 2 diabetes.

A strength of the study is the use of a well-documented and reproducible model of nutritional insult in utero to determine the short- and long-term impacts on β-cell mass, and changes to the mTOR pathway. A limitation of this approach is that the low-protein diet intervention is isocalorific with the control diet, the balance of calories being provided by additional carbohydrate. We cannot therefore separate the net effect of a substantial reduction in dietary protein from a modest increase on carbohydrate. A further limitation of the experimental plan is the inability to quantify separately the magnitude of changes in mTOR, Raptor and down-stream signaling components between the α-cell and β-cell compartments of the isolated islets of Langerhans. The alternative would have been to separate islets enzymatically into dispersed cell populations and separate the islet cell types using fluorescence-activated cell sorting prior to separate analysis of the mTOR pathway. A serious limitation of such an approach would be the likelihood that mRNA expression and protein synthesis would be substantially modified by the cellular stress of isolation and loss of cell–cell communication within islets.

Our hypothesis included the bias that exposure to a low-protein diet in utero would cause detectable changes in mTOR and STING presence and signaling predominantly in β-cells during early postnatal life, with long-term implications for metabolic control. Based on previous literature it was not anticipated that mTOR would be most strongly associated with α-cells rather than with β-cells, and that analysis of downstream signaling molecules would reflect the net effect of α- and β-cell activity. Further to this we assumed that changes in the mRNA expression of mTOR-associated signaling molecules in early postnatal life would predict longer-term changes in β-cell function alone. The experimental plan did not therefore include comparisons of mRNA expression of mTOR signaling molecules and activated protein presence at time points later than 7 days which, in retrospect, would have proved valuable given the presence of mTOR in α-cells also.

In conclusion, these studies show that long-term changes in α- and β-cell mass and function that result from exposure of mice to a LP diet in utero are associated with changes in the presence and activity of proteins contributing to the structure and actions of mTORC complexes apparent early in postnatal life. The impact of altered mTOR signaling at this time may predominantly influence the trajectory of α-cell mass and function since a lower life trajectory of β-cell mass has already been established. The implications of these changes are reflected in a decreased ability of the endocrine pancreas to compensate for increased metabolic stress in later life, and a failure to generate an adequate volume of functional β-cells during subsequent pregnancy leading to glucose intolerance [12].

## Figures and Tables

**Table 1:** Body weight, relative pancreatic weight and blood glucose levels of offspring from control or LP diet-fed mothers from age 1 to 130 days.

		Body Weight (g) (%) (mM)	Pancreas Weight/Body Weight	Blood Glucose
D 1	Control	1.61 ± 0.06	0.33 ± 0.06	4.1 ± 0.3
	LP	1.41 ± 0.04 *	0.35 ± 0.04	4.1 ± 0.2
D 7	Control	5.14 ± 0.38	0.19 ± 0.04	5.8 ± 0.4
	LP	4.47 ± 0.17 **	0.12 ± 0.03*	5.5 ± 0.3
D 30	Control	15.4 ± 0.7	0.81 ± 0.03	7.5 ± 0.5
	LP	14.4 ± 0.6	0.90 ± 0.14	7.4 ± 0.6
D 130	Control	25.6 ± 0.7	0.90 ± 0.08	6.8 ± 0.4
	LP	25.1 ± 0.5	0.93 ± 0.05	6.3 ± 0.3

* *p* < 0.05; ** *p* < 0.01 vs. control. Mean ± SEM; *n* = 15 − 24 for body weight and *n* = 6 for blood glucose.

## Supplementary Materials

The following are available online at https://www.mdpi.com/2072-6643/11/3/605/s1, Figure S1: Representative western blots from Figure 1B showing the abundance of mammalian target of rapamycin (mTOR) protein in isolated islets relative to β-actin at 7, 30 and 130 days. Figure S2: Immunohistochemical localization of mTOR in an islet from a 130-day-old control diet-fed mouse. Figure S3: Relative changes in phosphorylated mTOR activity determined by western blot relative to β-actin in the α−cell line αTC1-6 or the β-cell line MIN6 cells. Figure S4: Effect of rapamycin on cell proliferation and apoptosis is isolated pancreatic islets from a 30-day old mouse that had received control diet. Figure S5: Immunofluorescence localization of STING in a mouse islet from a 30-day old control diet-fed mouse. Table S1: Composition of the isocaloric LP and control diets. Table S2: Primer source and amplicon sizes for primers utilized for quantitative real-time PCR analysis, and forward and reverse primer sequences for TSC2 and proglucagon.
