# Supplementary material for: Offspring of Mice Exposed to a Low-Protein Diet in Utero Demonstrate Changes in mTOR Signaling in Pancreatic Islets of Langerhans, Associated with Altered Glucagon and Insulin Expression and a Lower β-Cell Mass
Source: Nutrients. 2019 Mar 12;11(3):605. doi: 10.3390/nu11030605 (PMC6471519; doi:10.3390/nu11030605)
Supplement: Supplementary file 1 [file nutrients-11-00605-s001.zip › Supplementary files/Supplementary Figures/Supplementary Figure Legends.docx]

**Supplementary Figure S1.** Representative western blots from Figure 1B showing the abundance of mammalian target of rapamycin (mTOR) protein in isolated islets relative to β-actin at 7, 30 and 130 days. Western blots for islets from two separate mice in each lane at each of days 7, 30 and 130 age are shown. These represent the results from two of the six animals in each group shown in Figure 1B. The presence of pmTOR is shown for animals previously exposed in utero to control diet (C) or low-protein diet (LP) relative to the abundance of β-actin. The positions of the relevant protein molecular weight ladder markers are shown to the right.

**Supplementary Figure** **S2.** Immunohistochemical localization of mTOR in an islet from a 130-day-old control diet-fed mouse using DAB chromogen. Staining is located in the α−cell-rich periphery of the islet. Scale bar represents 50 μm.

**Supplementary Figure S3. A)** Relative changes in phosphorylated mTOR activity determined by western blot relative to β-actin in the α−cell line αTC1-6 or the β-cell line MIN6 cells following incubation for 72h in the presence or absence of rapamycin (20 or 100 nM). Results are expressed as mean ± SEM relative to control incubations without rapamycin; n=3-4, **p* <0.05 vs. control. B) Representative duplicate images are shown for western blots for one experiment each with αTC1-6 or MIN6 cells. The positions of the relevant protein molecular weight ladder markers are shown.

**Supplementary Figure S4.** Effect of rapamycin on cell proliferation and apoptosis is isolated pancreatic islets from a 30-day old mouse that had received control diet. Islets were isolated from offspring of 30-day-old mice that received control diet and cultured for 72h in the presence of rapamycin (50 or 100 nM). Cell proliferation was assessed by labeling with EdU (nuclear antigen of DNA replication) (1μM) for 6h prior to fixation for immunofluorescence staining. Panel A shows EdU^+^ cells (red) and insulin (green) with cell nuclei (DAPI (4,6-diamidino-2 phenylindole), blue) for an islet in the absence of rapamycin. The number of EdU-positive/insulin-positive cells as a percentage of total insulin-positive cells following incubation with rapamycin are shown in panel B. The presence of apoptosis is shown in panel C by terminal deoxynucleotidyl transferase (TdT) dUTP Nick-End Labeling (TUNEL) assay (red) relative to the presence of insulin (green). The percent of apoptotic insulin-positive cells relative to total insulin-positive cells following incubation with rapamycin is shown in D. Values show Mean ± standard deviation (SD); **p* <0.01 vs. control for between 15-20 islets from each of six separate animals. Size scale bars are shown in A and C.

**Supplementary Figure** **S5**. Immunofluorescence localization of STING in a mouse islet from a 30 day old control diet-fed mouse. The section is stained for glucagon (red), STING (green) and insulin (light blue). Nuclei are stained using DAPI (dark blue) in C. STING was seen to co-localize with insulin in β−cells. Scale bars represent 50 μm.
