# Supplementary material for: Offspring of Mice Exposed to a Low-Protein Diet in Utero Demonstrate Changes in mTOR Signaling in Pancreatic Islets of Langerhans, Associated with Altered Glucagon and Insulin Expression and a Lower β-Cell Mass
Source: Nutrients. 2019 Mar 12;11(3):605. doi: 10.3390/nu11030605 (PMC6471519; doi:10.3390/nu11030605)
Supplement: Supplementary file 1 [file nutrients-11-00605-s001.zip › Supplementary files/Supplementary Tables.pdf]

Supplementary Table 1. Composition of the isocaloric LP and control diets (g/100g of diet).

	Control	LP
Cornstarch	40	40
Casein (88% protein)	22.3	8.6
Maltodextrin	13.2	13.2
Sucrose	10.0	23.6
Soybean oil	4.5	4.5
Cellulose	5.0	5.0
Mineral mix	3.5	3.5
Vitamin mix	1.0	1.0
L-Cysteine	0.3	0.3
Choline Bitartrate	0.25	0.25
Tert-butyl hydroquinone	0.0014	0.0014
DL-Methionine	2	0.8

Obtained from Bioserv, Frenchtown, NJ, USA

Supplementary Table 2. Primer source and amplicon sizes for TaqMan primers utilized for quantitative real-time PCR analysis, and forward and reverse primer sequences for TSC2 and proglucagon.

Target	Manufacturer	Catalogue #	Amplicon Size
mTOR	Applied Biosystems	Mm00444968_m1	65
Raptor	Applied Biosystems	Mm01242613_m1	64
Rictor	Applied Biosystems	Mm01307318_m1	75
Ins1	Applied Biosystems	Mm01950294_s1	80
Sting	Applied Biosystems	Mm01158117_m1	104
Gapdh	Applied Biosystems	Mm99999915_g1	109
		**Sequence**	
TSC2	Sigma-Aldrich	Forward AGGCCAGAGCAGCAGTG<br>Reverse AAGCCGACCCAGGCCTGTCA	
Proglucagon	Sigma-Aldrich	Forward AGCTTGGCCCAGGACACACT<br>Reverse CCAGCTGCCTTGCACCAGCA	
