# Supplementary material for: Offspring of Mice Exposed to a Low-Protein Diet in Utero Demonstrate Changes in mTOR Signaling in Pancreatic Islets of Langerhans, Associated with Altered Glucagon and Insulin Expression and a Lower β-Cell Mass
Source: Nutrients. 2019 Mar 12;11(3):605. doi: 10.3390/nu11030605 (PMC6471519; doi:10.3390/nu11030605)
Supplement: Supplementary file 1 [file nutrients-11-00605-s001.zip › Supplementary files/Supplementary Tables.docx]

**Supplementary Table 1.** Composition of the isocaloric LP and control diets (g/100g of diet).

Control LP

_____________________________________________________________________________

Cornstarch 40 40

Casein (88% protein) 22.3 8.6

Maltodextrin 13.2 13.2

Sucrose 10.0 23.6

Soybean oil 4.5 4.5

Cellulose 5.0 5.0

Mineral mix 3.5 3.5

Vitamin mix 1.0 1.0

L-Cysteine 0.3 0.3

Choline Bitartrate 0.25 0.25

Tert-butyl hydroquinone 0.0014 0.0014

DL-Methionine 2 0.8

Obtained from Bioserv, Frenchtown, NJ, USA

**Supplementary Table 2.** Primer source and amplicon sizes for TaqMan primers utilized for quantitative real-time PCR analysis, and forward and reverse primer sequences for TSC2 and proglucagon.

**_______________________________________________________________________________**

**Target Manufacturer Catalogue # Amplicon Size**

mTOR Applied Biosystems Mm00444968_m1 65

Raptor Applied Biosystems Mm01242613_m1 64

Rictor Applied Biosystems Mm01307318_m1 75

Ins1 Applied Biosystems Mm01950294_s1 80

Sting Applied Biosystems Mm01158117_m1 104

Gapdh Applied Biosystems Mm99999915_g1 109

**Sequence**

TSC2 Sigma-Aldrich Forward AGGCCAGAGCAGCAGTG

Reverse AAGCCGACCCAGGCCTGTCA

Proglucagon Sigma-Aldrich Forward AGCTTGGCCCAGGACACACT

Reverse CCAGCTGCCTTGCACCAGCA

________________________________________________________________________________
