# Supplementary figures and images for: Offspring of Mice Exposed to a Low-Protein Diet in Utero Demonstrate Changes in mTOR Signaling in Pancreatic Islets of Langerhans, Associated with Altered Glucagon and Insulin Expression and a Lower β-Cell Mass
Source: Nutrients. 2019 Mar 12;11(3):605. doi: 10.3390/nu11030605 (PMC6471519; doi:10.3390/nu11030605)

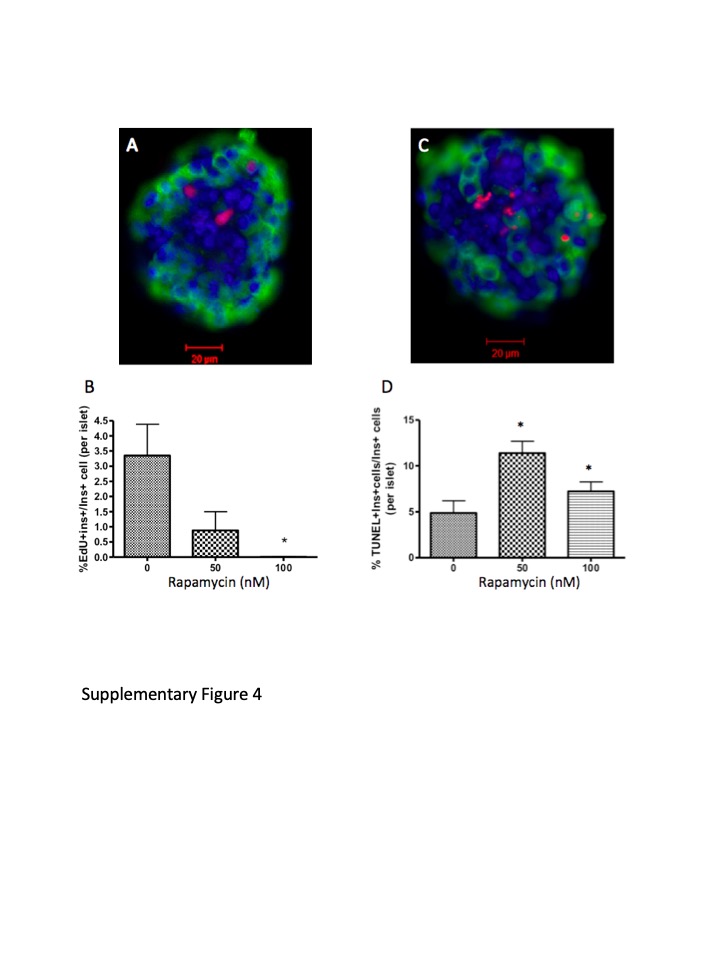

Supplement: Supplementary file 1 [file nutrients-11-00605-s001.zip › Supplementary files/Supplementary Figures/Supplementary Fig S4.jpeg]

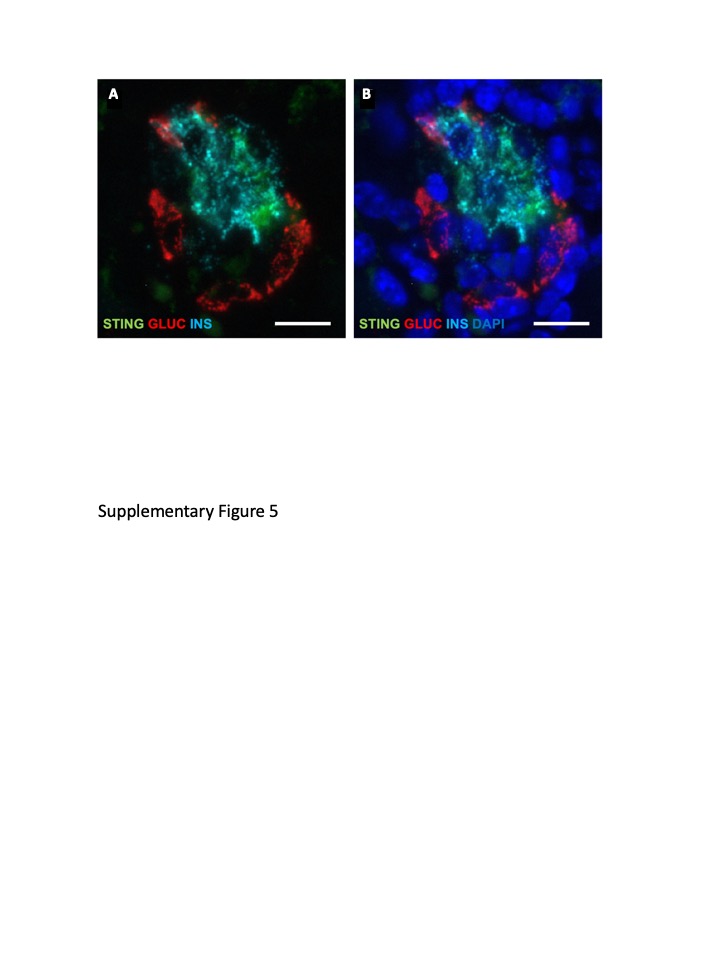

Supplement: Supplementary file 1 [file nutrients-11-00605-s001.zip › Supplementary files/Supplementary Figures/Supplementary Fig S5.jpeg]

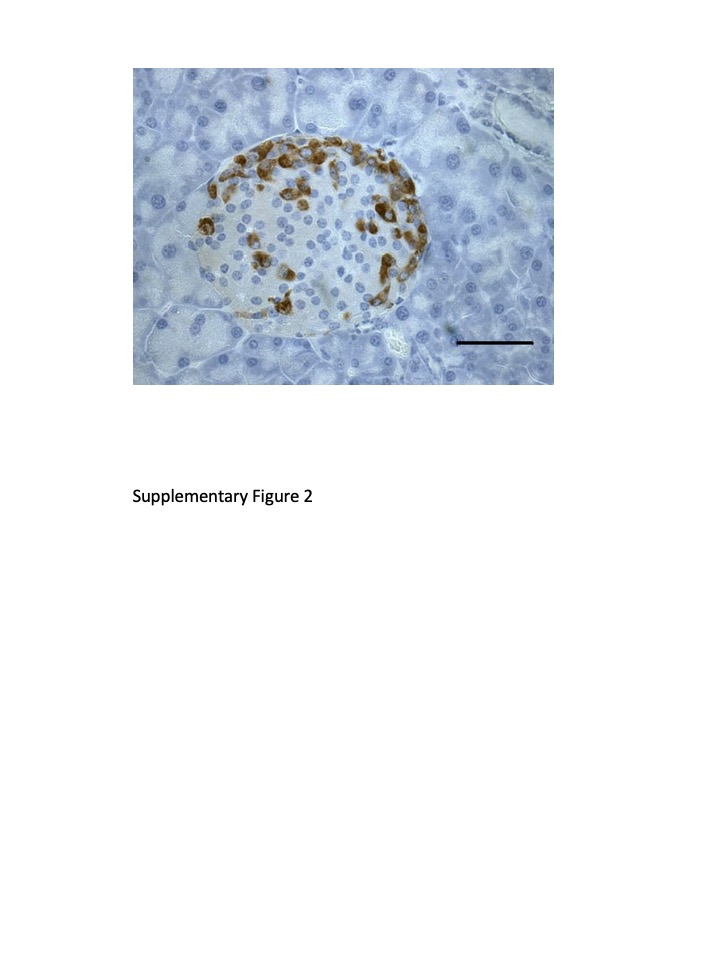

Supplement: Supplementary file 1 [file nutrients-11-00605-s001.zip › Supplementary files/Supplementary Figures/Supplementary Fig S2.jpeg]

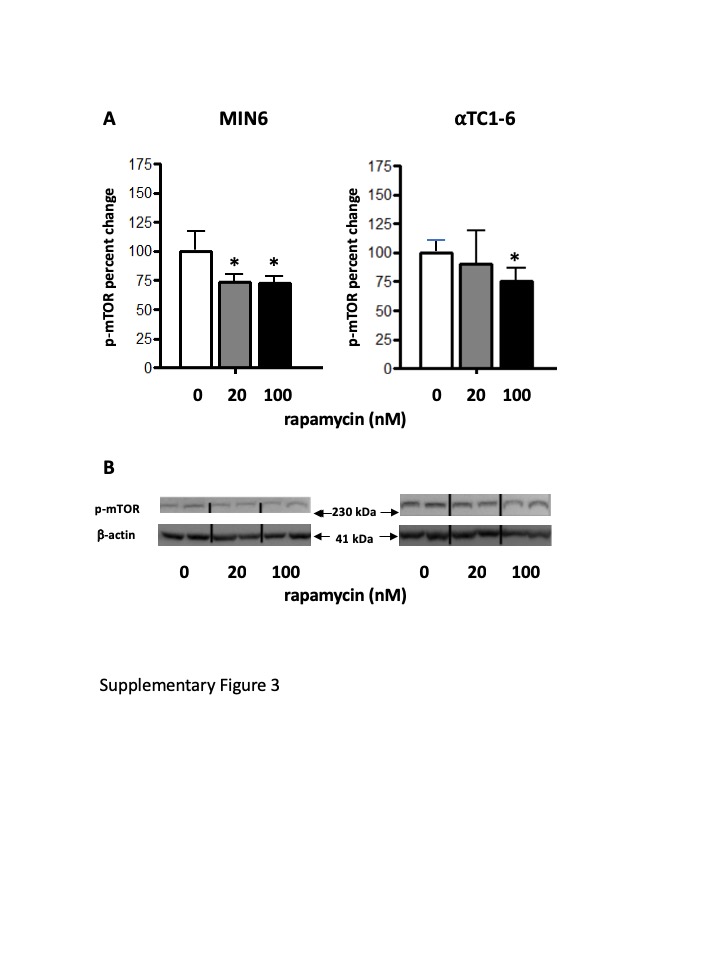

Supplement: Supplementary file 1 [file nutrients-11-00605-s001.zip › Supplementary files/Supplementary Figures/Supplementary Fig S3.jpeg]

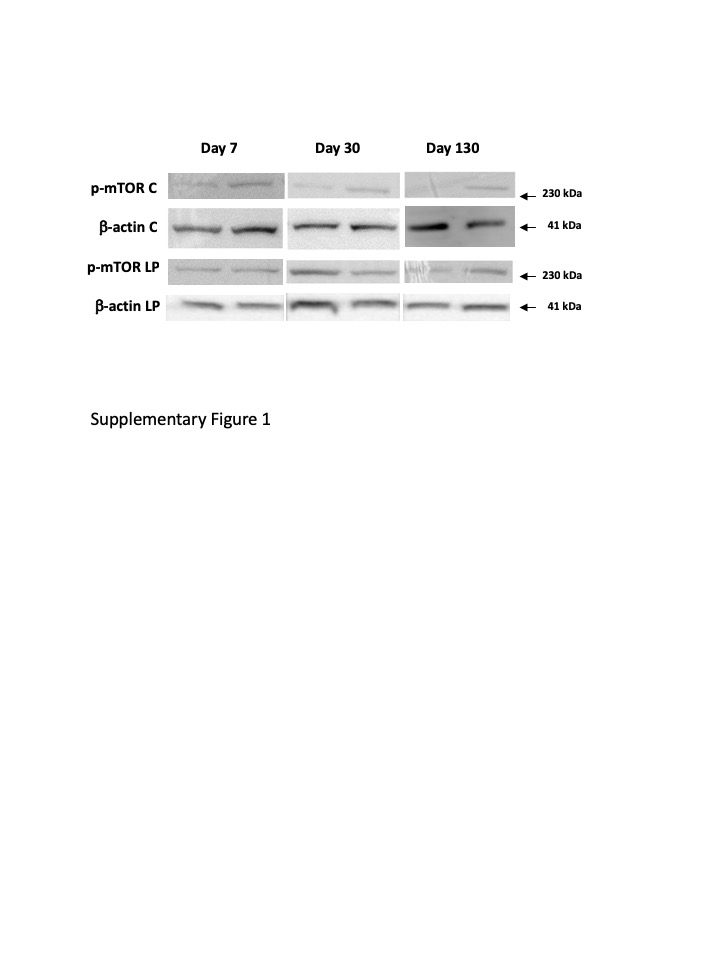

Supplement: Supplementary file 1 [file nutrients-11-00605-s001.zip › Supplementary files/Supplementary Figures/Supplementary Fig S1.jpeg]
